# Rates of Lipoprotein(a) Oxidation Accelerate at Elevated Levels and Attenuate With Atorvastatin Active Metabolites Compared With Other Statins

**DOI:** 10.1016/j.jacbts.2026.101664

**Published:** 2026-08-24

**Authors:** Samuel C.R. Sherratt, Peter Libby, Deepak L. Bhatt, R. Preston Mason

**Affiliations:** a Mount Sinai Fuster Heart Hospital, Icahn School of Medicine at Mount Sinai, New York, New York, USA; b Elucida Research, Beverly, Massachusetts, USA; c Mass General Brigham, Harvard Medical School, Boston, Massachusetts, USA

**Keywords:** atorvastatin metabolites, lipoprotein(a), oxidation, statins

## Abstract

Lipoprotein(a) [Lp(a)] levels are associated with increased cardiovascular risk, possibly linked to its oxidized phospholipid content that may accelerate lipoprotein oxidation. Attenuating this process could reduce Lp(a) atherogenicity. Atorvastatin is metabolized into 2 hydroxylated metabolites, which account for low-density lipoprotein reduction and potential antioxidant activity. We compared these metabolites' effects to other active statins on oxidation of plasma samples enriched with various levels of Lp(a) or other apolipoprotein B particles, as well as lipid membranes enriched with cholesterol. Lp(a) oxidized more rapidly than other apolipoprotein B particles and accelerated in a nonlinear fashion at levels associated with cardiovascular risk. The metabolites inhibited oxidation of each fraction in contrast to other statins and consistent with cholesterol-enriched membranes. The metabolites' unique phenoxy group facilitated membrane partitioning, leading to free radical scavenging activity as compared with other statins. The antioxidant actions of atorvastatin metabolites may reduce the atherogenicity of Lp(a) in patients independent of their levels.

## Highlights

• Enriched fractions of Lp(a) oxidized more rapidly in a nonlinear fashion.
• Lp(a)-enriched fractions underwent more rapid oxidation than matching enrichments containing other ApoB particles (ie, sdLDL, LDL).
• Atorvastatin hydroxylated metabolites attenuated Lp(a) and other ApoB particle oxidation, whereas other statins had no effect.
• The antioxidant activity of atorvastatin hydroxylated metabolites was linked to their unique phenoxy moiety, which both increased membrane partitioning and facilitated free radical scavenging.
• Reduction in Lp(a) oxidation with active atorvastatin metabolites may reduce Lp(a)-associated atherogenicity irrespective of levels in patients.

## Visual Abstract

**Figure:**
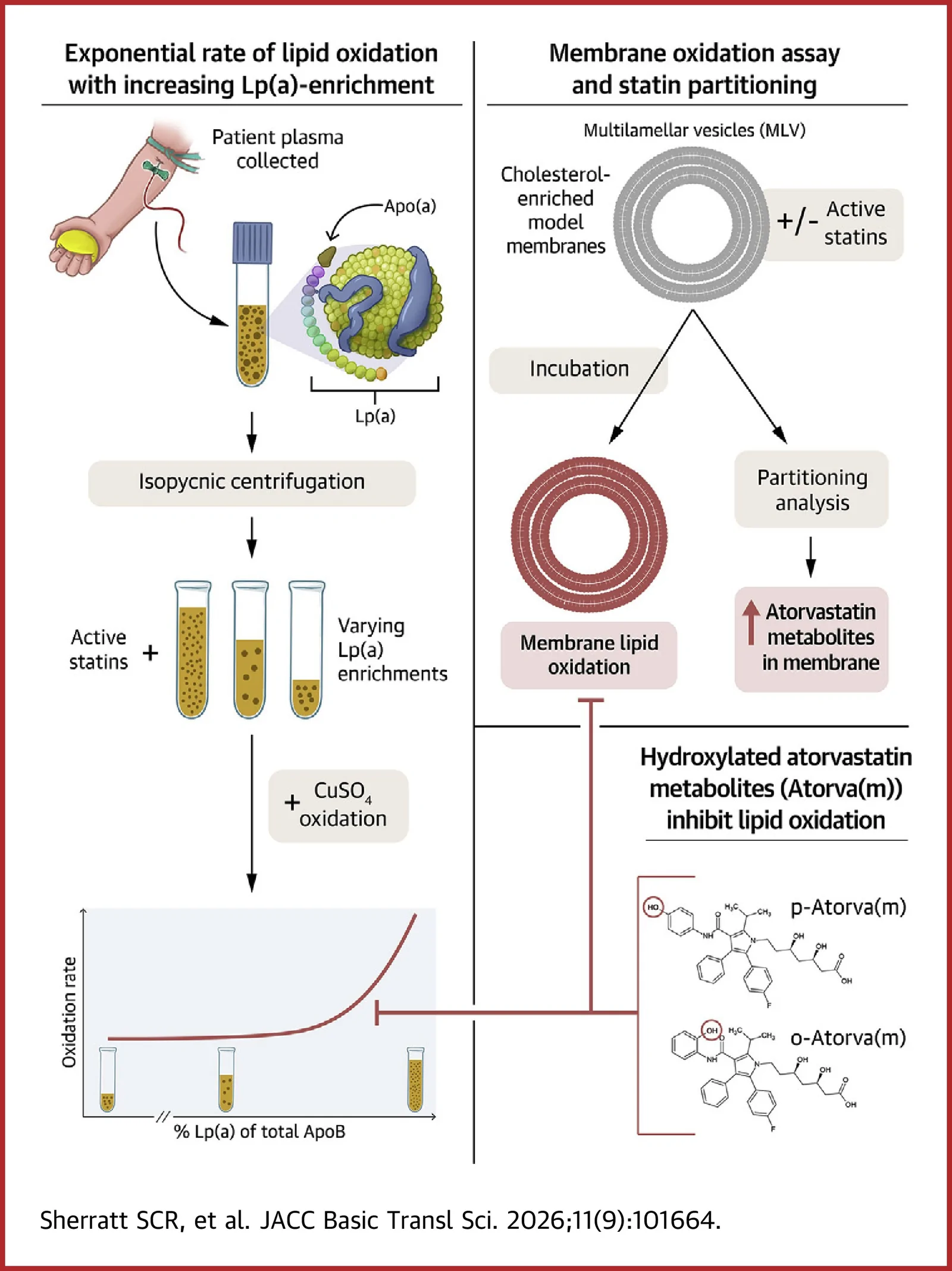

Lipoprotein(a), or Lp(a), is a low-density lipoprotein (LDL)-like lipid particle in which apolipoprotein B-100 (ApoB) is covalently linked to apolipoprotein(a) [apo(a)] via a disulfide bond.^1^ Circulating Lp(a) levels in patients are ∼90% determined by genetics, specifically the number of kringle IV type 2 (KIV-2) repeats in the apo(a) gene (*LPA*), which in turn dictates the size of apo(a), and widely varies among different ethnic populations.^2^ Elevated Lp(a) levels have been linked with atherosclerotic cardiovascular disease (ASCVD) as well as aortic valve stenosis and calcification.^3^, ^4^, ^5^, ^6^, ^7^ The atherogenic mechanisms of Lp(a), although incompletely understood, may be linked to the presence of oxidized phospholipid (oxPL) on Lp(a) as well as oxidation of intima-retained Lp(a) in atherosclerosis.^8^^,^^9^ We have previously shown that ApoB-lipoprotein fractions enriched with Lp(a) undergo more rapid oxidation in vitro than those enriched with LDL, TG-rich LDL (very-low-density lipoprotein) or even small dense LDL (sdLDL).^10^ In that study, the oxidized Lp(a) induced proinflammatory and stress response protein expression in human endothelial cells (ECs), including matrix metalloproteinase-1 and multiple heat shock proteins. Blunting Lp(a) oxidation, in turn, reversed these broad changes in protein expression.

Clinical investigations have shown that statin therapy is associated with changes in plasma Lp(a) levels, though the magnitude of change varies widely between studies.^11^, ^12^, ^13^, ^14^, ^15^ Large increases in Lp(a) levels (≥50 mg/dL), both at baseline and achieved on statin therapy, are associated with increased residual ASCVD risk.^11^^,^^13^^,^^14^ It is believed that any increase in Lp(a) with statin therapy is due to increased *LPA* expression in liver cells, leading to enhanced apo(a) synthesis.^12^ Given these complex effects of statins on Lp(a) levels and potential implications for ASCVD risk determination, it is important to understand their effects on Lp(a) atherogenicity and quality, including its susceptibility and rate of oxidative modification as compared with other ApoB-containing particles.

Atorvastatin is extensively metabolized to 2-*ortho*- and 4-*para*-hydroxylated derivatives along with various β-oxidation products by cytochrome P450 3A4 (CYP3A4) metabolism (Supplemental Figure 1). Approximately 70% of LDL reduction (3-hydroxy-3-methyl-glutaryl-coenzyme A [HMG-CoA] reductase inhibition) is due to the equipotent 2-*ortho* and 4-*para* hydroxylated metabolites (*o*-Atorva(m) and *p*-Atorva(m), respectively) that are prevalent in circulation.^16^^,^^17^ This is in contrast to other statins like pravastatin and rosuvastatin, which are neither metabolized into active metabolites nor that share these chemical properties.^18^ We have previously demonstrated that *o*-Atorva(m) inhibited LDL and membrane oxidation through a novel and direct antioxidant effect not replicated with other statins, and the antioxidant benefits of *o*-Atorva(m) extended to different-sized ApoB-containing particles.^19^ These findings suggest that the benefits of *o*-Atorva(m) are associated, in part, with its unique physicochemical properties and potent antioxidant activity.

Whether the reported antioxidant effects of atorvastatin metabolites extend to its other active hydroxylated metabolite and in Lp(a)-enriched lipoprotein fractions, even at elevated levels, remains unknown and served as the basis for the current study. We hypothesized that the antioxidant capacity of the active hydroxylated metabolites of atorvastatin would be superior to the other statins—namely rosuvastatin, simvastatin, pravastatin, and pitavastatin—in the more-rapidly oxidizing Lp(a)-enriched fractions as well as other atherogenic ApoB-containing particles and in cholesterol-enriched phospholipid membranes.

## Materials and Methods

### Materials

**Figure 1:**
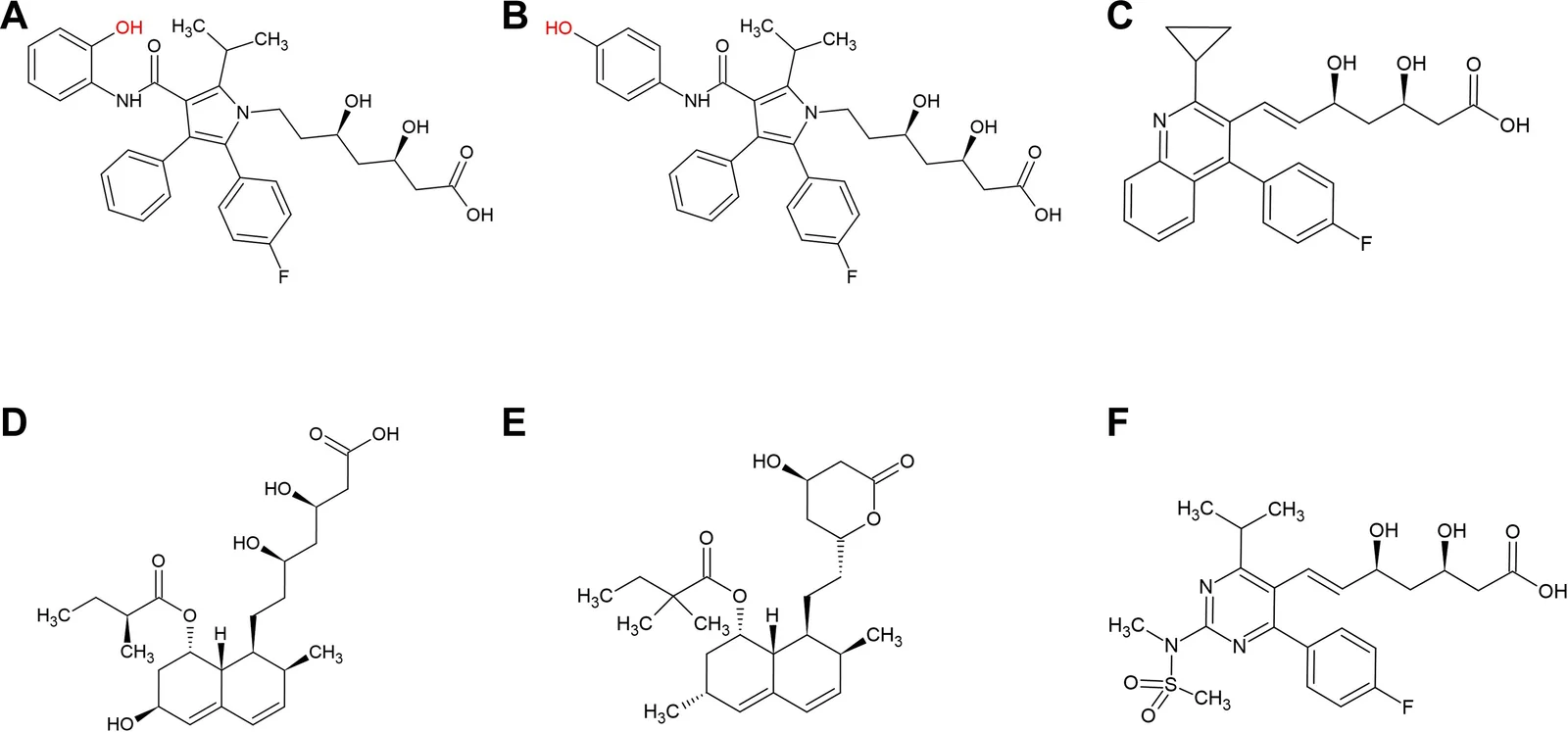
Chemical Structures of Statins Investigated in This Study (A) *o*-Atorva(m), (B) *p*-Atorva(m), (C) pitavastatin, (D) pravastatin, (E) simvastatin, (F) rosuvastatin. Hydroxyl groups on atorva (m) highlighted in red. Structures created using ChemSketch (Freeware) version 2024.2.3 from Advanced Chemistry Development (ACD)/Labs.

All lipoprotein fractions were anonymized and obtained from a commercial vendor (Kalen Biomedical LLC). Institutional Review Board approval was therefore not required for this study. Enriched fractions of Lp(a) (41%-75% of total ApoB), sdLDL, and LDL were pooled from the plasma of multiple (n = 2-3) healthy donors and then isolated by isopycnic centrifugation using OptiPrep media as previously described.^20^ The *o*-Atorva(m) was purchased from Toronto Research Chemicals, while *p*-Atorva(m), atorvastatin parent (atorva), rosuvastatin (rosuva), simvastatin (simva), pravastatin (prava), and pitavastatin (pitava) were purchased from Cayman Chemicals. All statins were solubilized in ethanol or methanol to 1.0 mM and stored at −20 °C. 1,2-dilinoleoyl-sn-glycero-3-phosphatidylcholine (DLPC), 1-palmitoyl-2-oleoyl-sn-glycero-3-phosphocholine (POPC), and cholesterol (from ovine wool) were purchased from Avanti Polar Lipids. All colorimetric reagents were purchased from Sigma-Aldrich. The chemical structures of each statin are shown in Figure 1.

### Lp(a)-enrichment oxidation

Oxidation of the various Lp(a)-enriched fractions was carried out based on previously published methods.^10^ The enriched fractions contained the indicated percentage of Lp(a) (based on total ApoB protein), with the remaining content composed of LDL. We used a 0% enriched fraction (containing 100% LDL) as a reference. Each fraction (0%-75%) was delivered at an equivalent total protein concentration (50 μg/mL) and incubated in the absence or presence of *o*-Atorva(m) (10 μM) or equivolume methanol vehicle for 30 minutes in a lightly shaking water bath set to 37 °C. Oxidation was initiated using 20 μM copper sulfate. At multiple time points 0 to 2 hours, 100 μL aliquots were removed from each sample and added to separate test tubes for colorimetric analysis. To each aliquot, we added 1.0 mL thiobarbituric acid, 10 μL trichloroacetic acid, 10 μL butylated hydroxytoluene, and 10 μL ethylenediaminetetraacetic acid. Samples were then incubated for 30 minutes at 100 °C. Concentrations of malondialdehyde (MDA) were detected using ultraviolet-visible (UV/Vis) spectrophotometry at 532 nm, and nonspecific background scattering was collected at 600 nm for correction. Comparative rates of oxidation were determined based on the percentage of total oxidizable lipid at 0.5 hour as previously described.^10^

### Lp(a), sdLDL, and LDL oxidation ± statins

Oxidation analyses of enrichment Lp(a), sdLDL, and LDL fractions were carried out as described in the previous section. Each lipoprotein fraction was delivered at equivalent total protein concentration (50 μg/mL). We analyzed dose-dependent effects of *o*-Atorva(m) from 1.0 to 10.0 μM as well as the effects of equimolar statin (10 μM). *p*-Atorva(m) and the combination of *p*- + *o*-Atorva(m) were also analyzed with the total concentration of atorvastatin metabolites equivalent to the concentrations of the separate treatments. Rates of oxidation of the different lipoprotein fractions were again described by the percentage of total oxidized lipid measured from 0 hour to peak oxidation time (defined as the time point corresponding to the highest levels of MDA measured in vehicle-treated controls). Antioxidant actions of the different statins were compared by changes in MDA levels over time, with statistical comparisons performed at the peak oxidation time.

### Membrane oxidation analysis ± statins

We also analyzed the combined effects of the atorva(m) vs other statins in model membranes with elevated cholesterol content using previously described methods.^20^ Briefly, multilamellar vesicles (MLVs) were prepared as mixtures of DLPC and cholesterol at a 0.6:1 cholesterol-to-phospholipid mole ratio, along with each statin (10 μM total) or equivolume vehicle. MLV components were vortex mixed under a stream of nitrogen gas to remove residual solvent. This was then followed by incubation in a vacuum desiccator >1 hour. The components were resuspended in a N-[2-hydroxyethyl]piperazine-N′-[2-ethanesulfonic acid] (HEPES) diffraction buffer (0.5 mM HEPES,154 mM NaCl, pH 7.3) warmed to room temperature to a final phospholipid concentration of 1.0 mg/mL. The samples were then vigorously perturbed via vortex mixer for 3 minutes to form the MLVs. The samples were then placed in a lightly shaking water bath set at 37 °C. At 24-hour intervals between 0 and 72 hours, aliquots were removed and analyzed for primary lipid oxidation products (lipid hydroperoxide, LOOH) using a iodide-based colorimetric reagent as previously described.^20^

### Membrane partitioning analysis

We compared the membrane partitioning (*Kp*_*mem*_) of representative lipophilic (*o*-Atorva(m), atorva, pitava) and hydrophilic (rosuva) statins using a novel UV/Vis spectrophotometric method. Model membranes were prepared as mixtures of POPC and cholesterol at a 0.3 cholesterol-to-phospholipid mole ratio. In addition to the lipid components, each compound was added at 30 μM. This concentration was chosen based on standard curves that were created in the absence of membranes, which yielded ideal absorbance values to observe maximal partitioning and linearity. Each component was added to borosilicate test tubes and mixed via vortex under a steady stream of nitrogen. Samples were then placed in a desiccator for ≥1 hour to remove residual solvent. HEPES buffer was added to yield a total phospholipid content of 250 μg in each sample. MLVs were created by vortex mixing the samples for 3 minutes. The membrane solutions were then centrifuged for 1 hour at 5 °C to separate MLVs from supernatants, which were then removed and analyzed using UV/Vis spectrophotometry scanning from 190 to 500 nm. The absorbance value at the statin-specific analytical wavelength - based on their respective standard curves - was then recorded. The concentration of the agents in each component (membrane or buffer supernatant) was determined based on the absorbance value in the supernatant (*i.e.*, Starting Concentration − Concentration in Supernatant = Concentration in Lipid). Corrections for nonspecific binding and background scattering were included in preparation of the standard curves and in each measurement, respectively. The concentration of each compound in the supernatant was then used to determine the concentration of each compound in the membranes, and this information was used to determine the *Kp*_*mem*_ based on the following formula:

$$Kp_{\left[mem\right]}=\frac{\frac{\mu g\;\text{Drug}\;\left(\text{in}\;\text{lipid}\right)}{\mu g\;\text{Lipid}}}{\frac{\mu g\;\text{Drug}\;\left(\text{in}\;\text{buffer}\right)}{\mu g\;\text{Buffer}}}$$

### Statistical analysis

Data are presented as the mean ± SD. All experimental conditions were tested using a sample size N = 3 to 9. Groups were compared using Student *t*-test or one-way analysis of variance with Tukey post hoc test for multiple pairwise comparisons. Normality was assessed using the Kolmogorov-Smirnov test. Analyses were performed using GraphPad InStat version 3.10 for Windows (GraphPad Software), and a *P* value <0.05 was considered statistically significant. Supporting data will be made available upon reasonable request to the corresponding author.

## Results

### Lp(a) enrichment and oxidation rate

**Figure 2:**
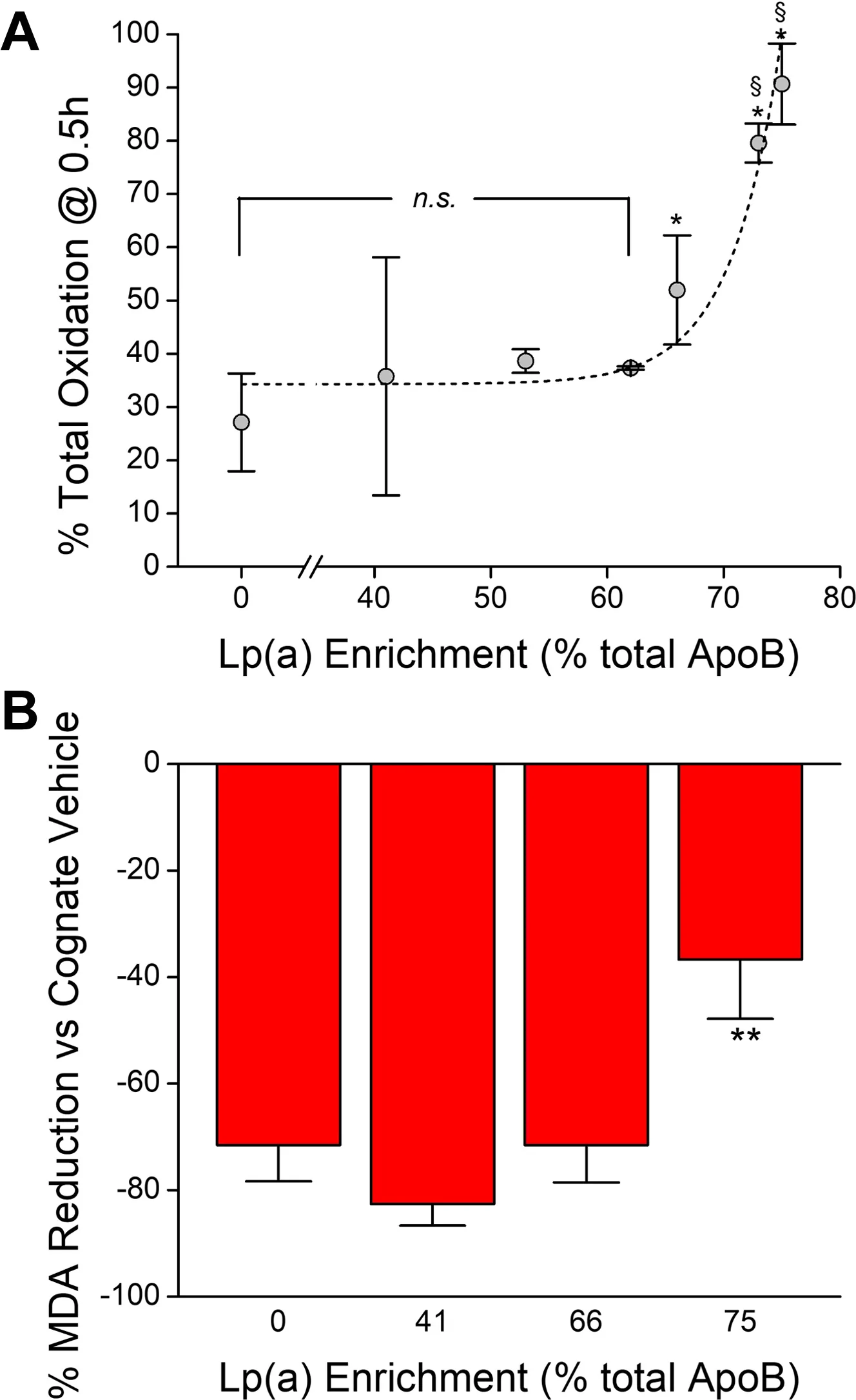
Effects of Lp(a)-enrichment on Oxidation Rate ± *o*-Atorva(m) (A) Effects of Lp(a)-enrichment (as % total apolipoprotein B [ApoB] protein) on rate of oxidation; (B) antioxidant effects of *o*-atorva(m) on Lp(a) oxidation at low, medium, and high enrichments. Rate of oxidation determined by dividing malondialdehyde (MDA) levels at 0.5 hour postcopper sulfate treatment by the peak MDA level captured (consistently measured at the 2 hour time point postcopper sulfate treatment). Statistical indicators: ∗*P* < 0.05 vs 0% to 53% enrichment; ^§^*P* < 0.05 vs62% and 66% enrichment (overall analysis of variance: *P* < 0.001). Values shown as mean ± SD (N = 3-9). (B) Values shown as % change of the peak MDA levels with 10 μM *o*-Atorva(m). Statistical indicators: ∗∗*P* < 0.01 vs other enrichments (overall analysis of variance *P* < 0.001). All tests were performed using the Tukey-Kramer multiple comparisons test. Values are shown as mean ± SD (N = 3).

We found a nonlinear relationship between the rate of oxidation of Lp(a)-enriched fractions and the Lp(a) content of those fractions at levels associated with increased cardiovascular risk (Figure 2A). From 0% Lp(a) through ∼60% Lp(a), the rate of oxidation at 0.5 hours was constant with no significant change. Above this enrichment, the rate of oxidation increased exponentially with the fastest rate of oxidation measured in the 75% enriched sample (91 ± 8% of peak MDA level reached by 0.5 h, *P* < 0.05 vs enrichments <70% Lp(a)). To determine whether the rate of oxidation impacted the effects of *o*-Atorva(m), we incubated the metabolite with 3 Lp(a) enrichments—low (41%), medium (66%), and high (75%)—as well as 100% LDL as a reference point. We observed consistent, >70% MDA reduction at 2 hour with *o*-Atorva(m) in 0%-66% Lp(a) enrichment, while the activity was slightly diminished in the highest Lp(a) enrichment (37%, *P* < 0.01 vs other enrichments, Figure 2B).

We then compared the rate of oxidation of the highest Lp(a) enrichment (75%) to a matching enrichment of sdLDL as well as nonenriched LDL. We found a significant trend in the rate of oxidation such that Lp(a) > sdLDL >LDL (Supplemental Figure 2). Baseline MDA levels were also higher in Lp(a) than the other fractions, which may indicate the presence of oxPLs as is characteristic of Lp(a).

### Effects of o-Atorva(m) vs other statins on lipoprotein oxidation

As ∼60% Lp(a) enrichment appeared to be the threshold for observing increased oxidation rate, we then compared the antioxidant effects of *o*-Atorva(m) to the other statins in “high-enriched Lp(a)” (>60% of total ApoB), “low-enriched Lp(a)” (<60% of total ApoB), sdLDL, and LDL. First, we observed consistent time- and dose-dependent reductions in MDA levels in all lipoprotein fractions with *o*-Atorva(m) (Supplemental Figures 3A to 3H). Significant reductions in MDA levels were observed early (0.5 hour) and sustained through 1 hour at all concentrations. By 2 to 3 hour, the time points at which we observed peak MDA levels produced in all vehicle-treated samples, the 5.0/10.0 μM treatments reduced MDA levels by 31%/37% in high-enriched Lp(a), 53%/57% in low-enriched Lp(a), 53%/62% in sdLDL, and 66%/72% in LDL, respectively (all *P* < 0.01 vs cognate vehicle).

**Figure 3:**
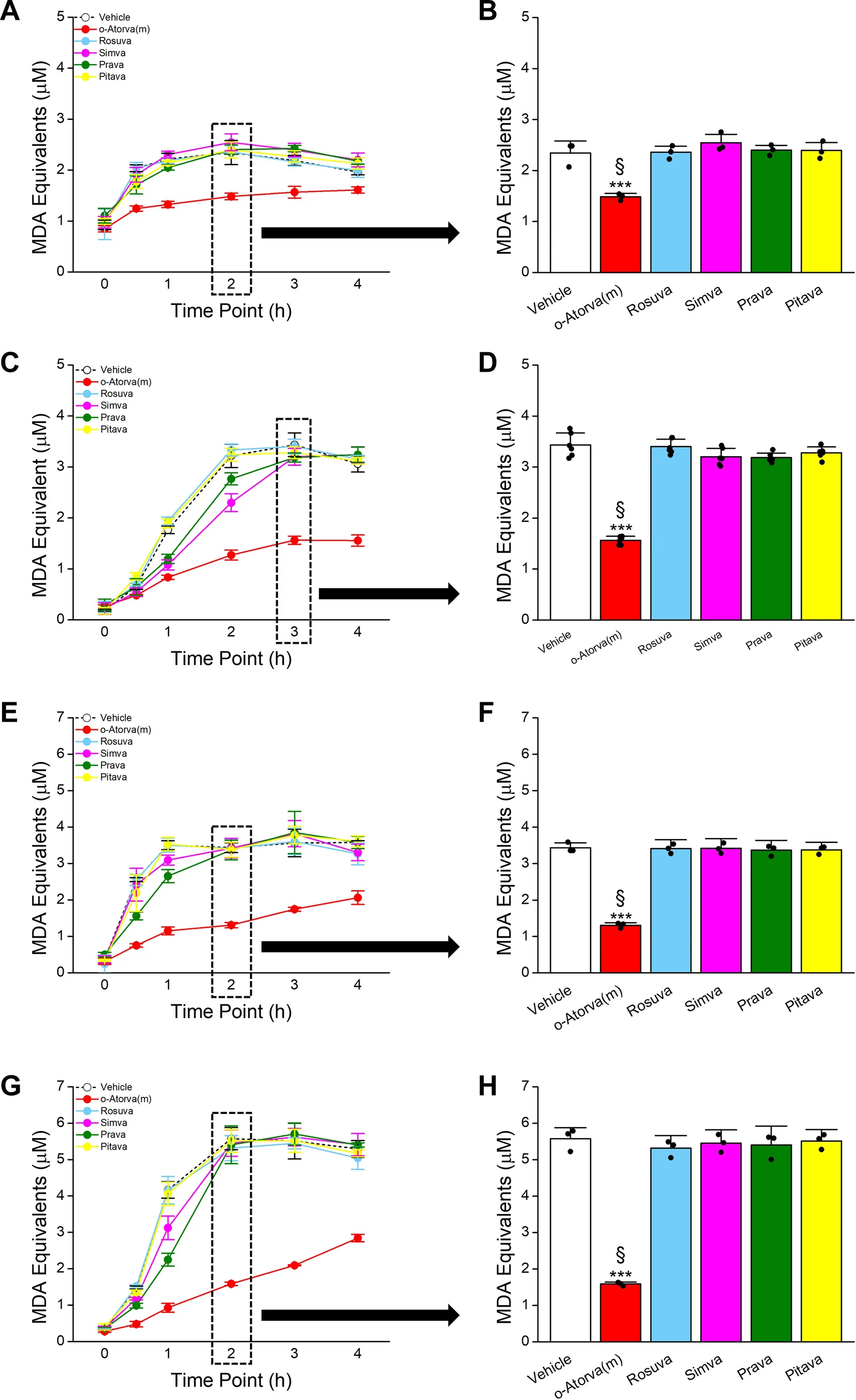
Comparative Effects of *o*-Atorva(m) vs Other Statins on Lipoprotein Oxidation Comparative effects of *o*-atorva(m) and other statins on oxidation of (A and B) high-enriched fractions of Lp(a) and (C and D) low-enriched fractions of Lp(a), (E and F) small dense low-density lipoprotein, and (G and H) low-density lipoprotein. Statistical indicators—∗∗∗*P* < 0.001 vs vehicle; ^§^*P* < 0.001 vs other statins. All tests were performed using the Tukey-Kramer multiple comparisons test; overall analysis of variance: *P* < 0.001 for each test. Values are shown as mean ± SD (N = 3-6). Individual data points represented by superimposed circles on each bar. MDA = malondialdehyde.

We then compared the effects of *o*-Atorva(m) to the other statins in each lipoprotein fraction (Figures 3A to 3H). Only *o*-Atorva(m) exhibited consistent, significant MDA reductions over time. At earlier time points, prava and simva exhibited slight antioxidant activity, albeit to a lesser extent than *o*-Atorva(m), and these effects largely disappeared by the peak vehicle-treated oxidation time, whereas the large MDA reductions with *o*-Atorva(m) persisted. We also tested whether the parent atorvastatin (atorva), which lacks the phenoxy moiety of its *ortho-* and *para-* hydroxylated metabolites, has antioxidant activity in LDL. There were no reductions in MDA at any time point with atorva compared with vehicle treatment (Supplemental Figure 4).

### Separate and combined antioxidant effects of o-Atorva(m) and p-Atorva(m)

**Figure 4:**
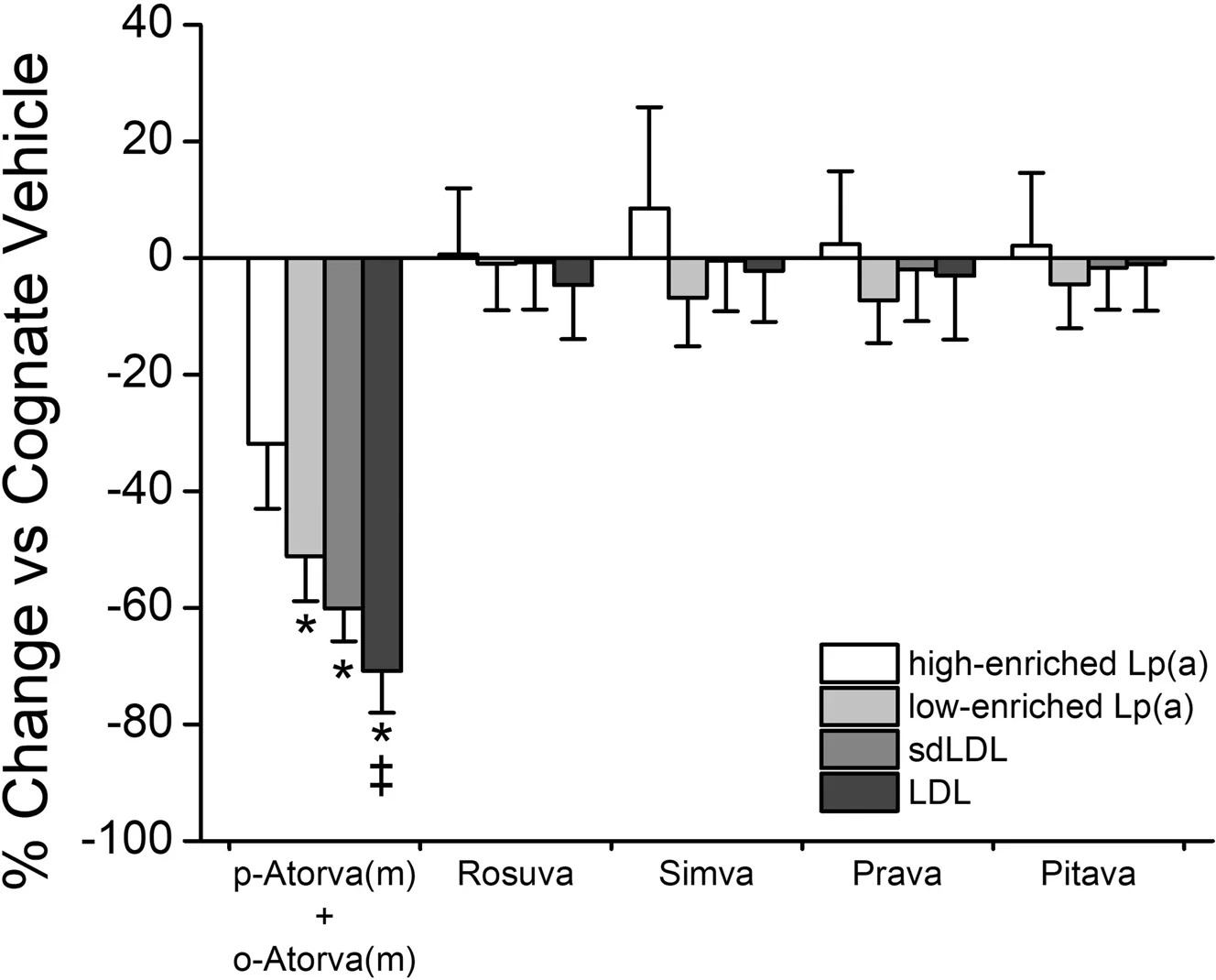
Comparative Effects of Combined Hydroxylated Atorvastatin Metabolites vs Other Statins Across Each Lipoprotein Fraction All treatments were delivered at the same concentration (10 μM total). Data calculated from the peak oxidation time point from each fraction. Statistical analyses performed between each lipoprotein fraction within each statin group. Statistical indicators: *p*- + *o*-Atorva(m): ∗*P* < 0.05 vs high-enriched Lp(a); ^‡^*P* < 0.05 vs low-enriched Lp(a) (overall analysis of variance: *P* < 0.001). Rosuva: (overall analysis of variance: *P* = 0.90). Simva: (overall analysis of variance: *P* = 0.30). Prava: (overall analysis of variance: *P* = 0.55). Pitava: (overall analysis of variance: *P* = 0.75). All tests were performed using Tukey-Kramer multiple comparisons test. Values are shown as mean ± SD (N = 3-6). Individual data points represented by superimposed circles on each bar. sdLDL = small dense low-density lipoprotein; LDL = low-density lipoprotein

We then evaluated the separate and combined effects of *p*-Atorva(m) and *o*-Atorva(m) in these lipoprotein fractions. We found consistent reductions in MDA levels for *p*-Atorva(m), *o*-Atorva(m), and their combination in each fraction (Supplemental Figure 5). Interestingly, *p*-Atorva(m) was slightly less effective than *o*-Atorva(m), indicating the location of the hydroxyl moiety may influence optimal intercalation into Lp(a) particles and/or free radical scavenging properties. At peak oxidation points, *p*-Atorva(m), *o*-Atorva(m), and the combination reduced MDA levels by, respectively, 18%, 37%, and 32% in high-enriched Lp(a), 45%, 55%, and 51% in low-enriched Lp(a), 51%, 62%, and 60% in sdLDL, and 70%, 72%, and 71% in LDL (all *P* < 0.05 vs cognate vehicle). When compared with the other statins, only the combination significantly reduced MDA levels in all lipoprotein fractions, with consistently greater reductions in MDA levels from LDL > sdLDL > low-enriched Lp(a) > high-enriched Lp(a) (Supplemental Figure 6, Figure 4).

**Figure 5:**
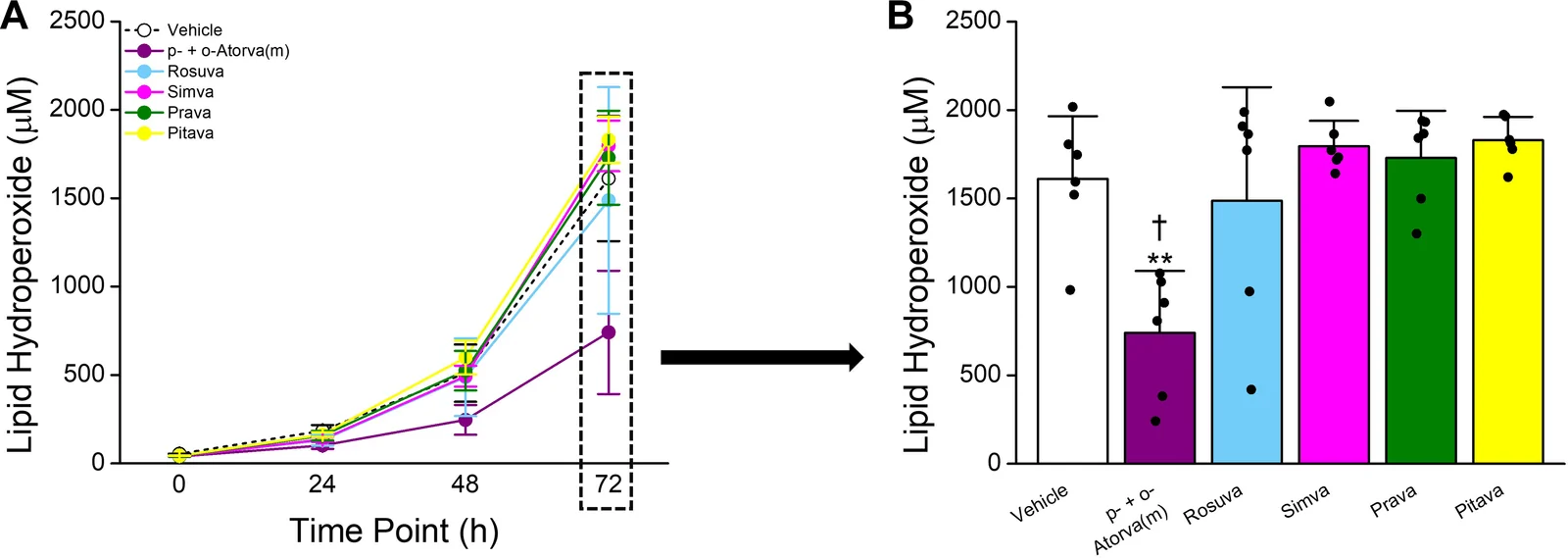
Comparative Effects of Combined Hydroxylated Atorvastatin Metabolites vs Other Statins on Membrane Oxidation (A) Comparative effects of combined hydroxylated atorvastatin metabolites vs other statins in model membranes containing elevated cholesterol through 72 hour and (B) at the 72-hour time point, corresponding to peak lipid hydroperoxide levels. All treatments were delivered at the same concentration (10 μM total). Statistical indicators: ∗∗*P* < 0.01 vs vehicle; ^†^*P* < 0.05 vs other statins (Tukey-Kramer Multiple Comparisons test; overall analysis of variance: *P* < 0.001). Values are shown as mean ± SD (N = 6). Individual data points represented by superimposed circles on each bar.

These effects were also observed in cholesterol-enriched model membranes (Figure 5). The combination reduced LOOH by 52% and 54% compared with vehicle-treated membranes after 48 hours and 72 hours (*P* < 0.01), respectively, whereas no other statin showed any antioxidant activity at any time point.

### Membrane partitioning of representative statins

**Figure 6:**
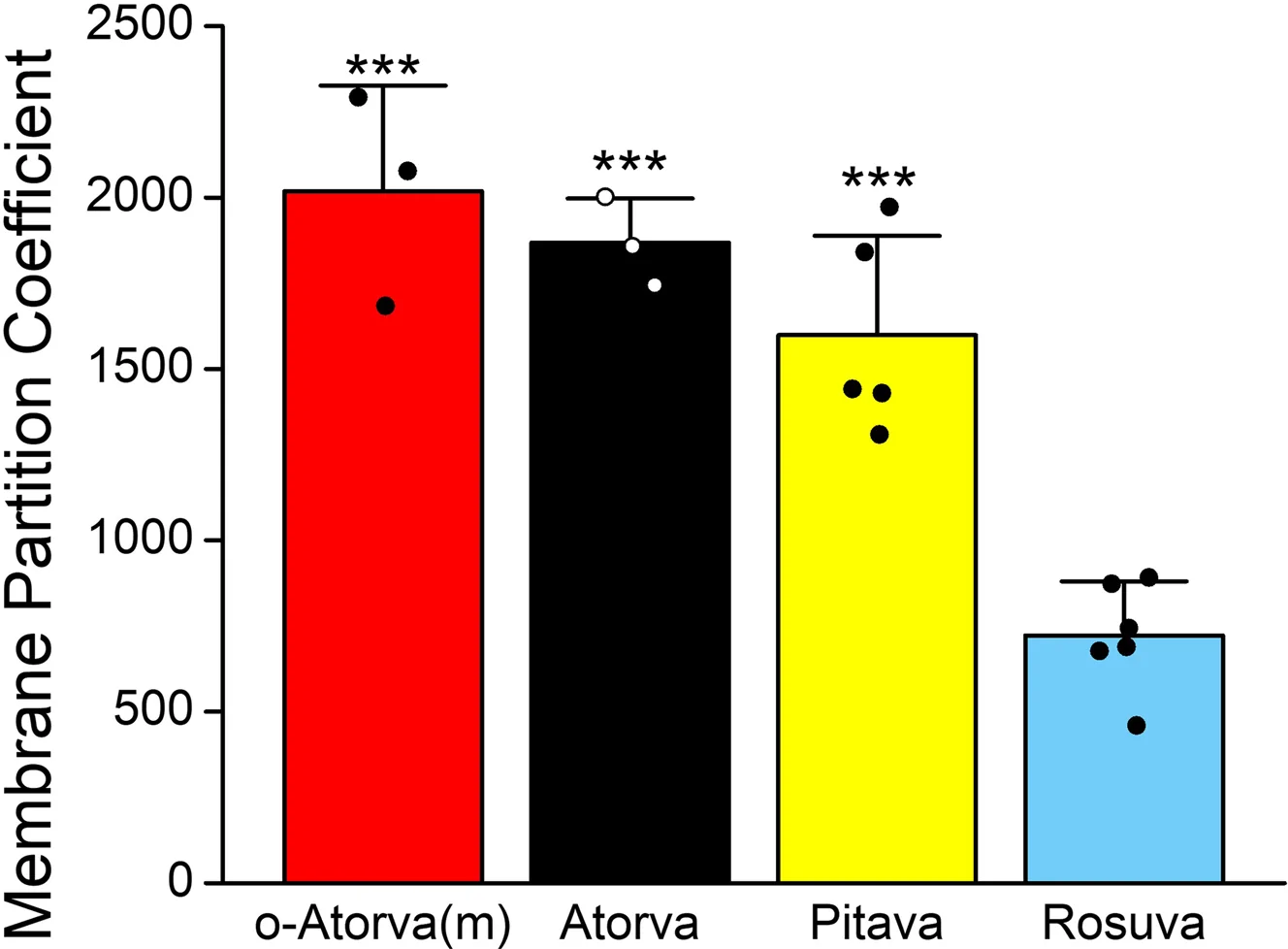
Membrane Partitioning of Representative Statins Statistical indicators: ∗∗∗*P* < 0.001 vs rosuva (Tukey-Kramer Multiple Comparisons Test; overall analysis of variance: *P* < 0.001). Values are mean ± SD (N = 3-6). Individual data points represented by superimposed circles on each bar.

We hypothesized that the superior lipid antioxidant activity of atorvastatin hydroxylated metabolites stems from the stable intercalation into lipid environments to allow for free radical scavenging by the phenoxy moiety. The antioxidant activity analyses support the latter portion of this hypothesis. To evaluate the first portion of the hypothesis, we compared the membrane partitioning of representative lipophilic and hydrophilic statins from our study. The lipophilic statins included *o*-Atorva(m), atorva, and pitavastatin, and the hydrophilic statin was rosuvastatin. We found that, as expected, all the lipophilic statins exhibited significant partitioning into model POPC membranes compared with rosuvastatin (Figure 6).

## Discussion

This study evaluated the antioxidant effects of multiple hydroxylated metabolites of atorvastatin compared with other statins in different lipid environments, including Lp(a) at various enrichments matched with other atherogenic ApoB lipoprotein fractions. For the first time, we described potent time- and dose-dependent antioxidant effects of these active hydroxylated atorvastatin metabolites in isolated Lp(a) across a range of Lp(a) levels. These effects were unique to the atorvastatin hydroxylated metabolites among the statins evaluated in this study. Oxidation analyses of other atherogenic ApoB particles and cholesterol-enriched model membranes showed similar trends, which is consistent with previously reported findings.^19^^,^^21^ We hypothesize that this unique effect of the atorvastatin metabolites depends on two key criteria: 1) stable penetration into lipid environments and 2) the presence of a phenoxy moiety which can participate in direct free radical scavenging. If either or both criteria are not met, we would not anticipate attenuation of lipoprotein or membrane oxidation with that statin.

We found, as expected, that multiple lipophilic statins—*o*-Atorva(m), atorva, and pitava—exhibit consistently high membrane partitioning compared with a representative hydrophilic statin (rosuva). Among the lipophilic statins, only the hydroxylated metabolites possessed the requisite chemistry to facilitate radical scavenging as shown in the current study and supported by quantum chemical analyses.^22^ The comparison between *o*-Atorva(m) and atorva is particularly striking, as the only difference between the chemical structures of the molecules is the addition of a hydroxyl group. Yet it is this hydroxyl group that transforms the molecule from an inactive to active—and potent—lipid antioxidant. Beyond this potent antioxidant action, these hydroxylated metabolites also account for the majority (∼70%) of the atorvastatin in circulation and resultant LDL-lowering actions. We may thus consider atorvastatin a “quasi-prodrug,” where the parent compound retains limited enzymatic antagonism with its metabolites providing most of the intended lipid-lowering benefits as well as pleiotropic actions, including broad lipoprotein/membrane antioxidant effects.^23^

**Figure 7:**
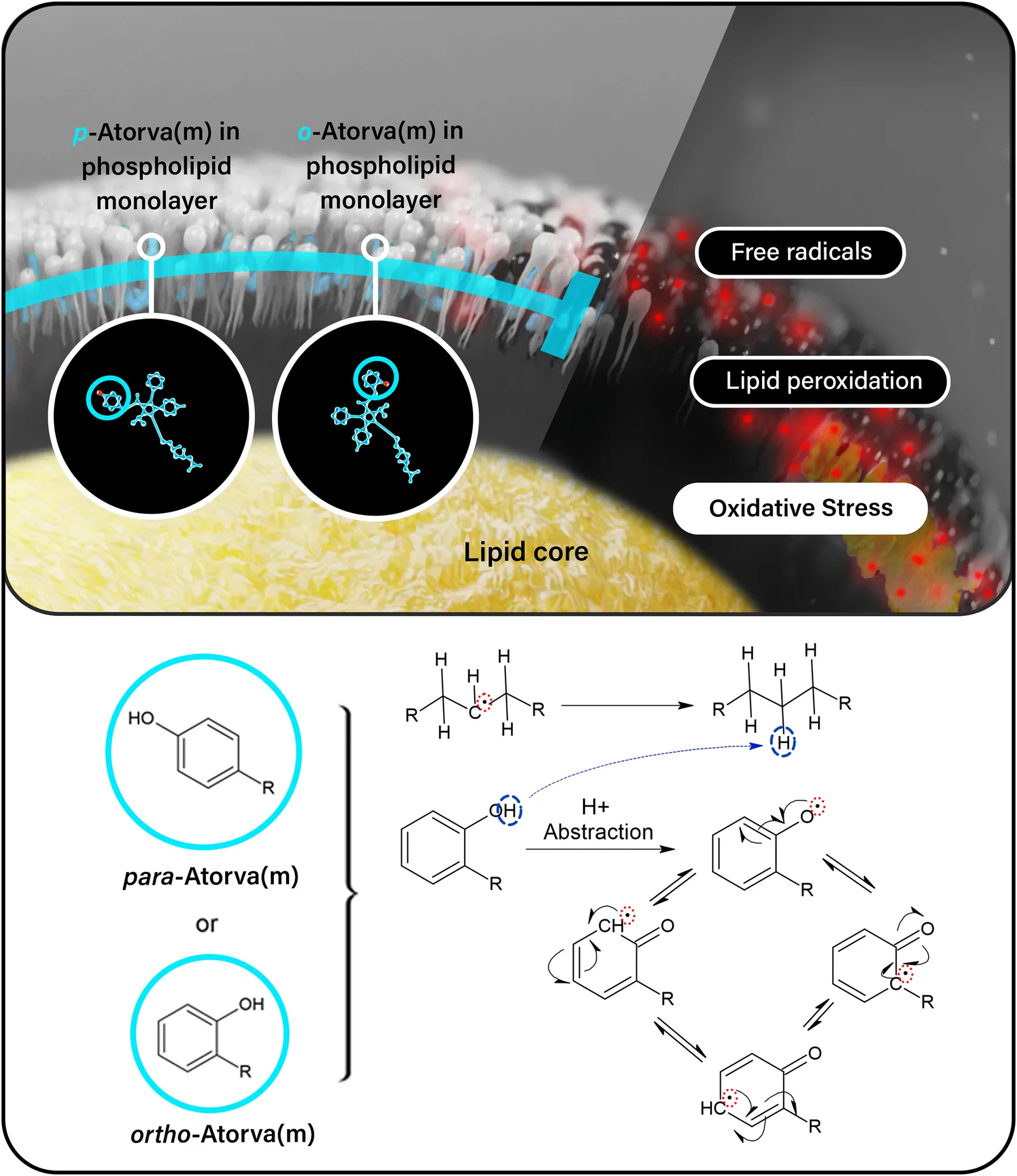
Proposed Mechanism for Antioxidant Activity of Hydroxylated Atorvastatin Metabolites in Lipoproteins The phenoxy group at both the ortho and para positions on atorvastatin gives rise to favorable electron density distribution to enable intercalation into the outer monolayer of lipoprotein particles. Here, the hydroxyl moiety can donate a hydrogen atom (blue circle) to stabilize free radicals, shown above as a lipid-centric radical (R-CH_2_-CH•-CH_2_-R, red circle). The resulting oxygen free radical (O•) on the atorvastatin metabolite can be stabilized by the delocalized electrons within the resonance structure of the adjacent aromatic ring.

These data are supported by previous x-ray diffraction studies in which we demonstrated that *o*-Atorva(m) partitions near the phospholipid headgroup and glycerol backbone interface of lipids, with its phenoxy group positioned in the central hydrocarbon core region in proximity to acyl chain unsaturated bonds vulnerable to oxidative modification.^21^ This location facilitates proton donation and resonance stabilization mechanisms for antioxidant benefits (see Figure 7). Briefly, the hydrogen atom on the hydroxyl group can be readily abstracted by adjacent carbon-centric radicals in these neighboring phospholipids. This stabilizes that radical but produces a phenoxy radical. The unpaired electron can then be stabilized by the delocalized electrons within the resonance structure of the adjacent aromatic ring. This effectively blunts the propagation of free radicals through the lipid environment, preventing further acyl chain and protein oxidation before eventual degradation. Previous studies in our laboratory have demonstrated these radical quenching actions of *o*-Atorva(m) in isolated ApoB-containing particles, in which the antioxidant actions were observed at low nanomolar concentrations in a dose-dependent manner.^19^ Oxidative modification of lipoproteins, especially LDL, has been extensively characterized^24^ and includes, among other things, increased uptake by scavenging receptors (eg, lectin-like oxidized LDL receptor-1, LOX-1) on ECs and macrophages with resulting cellular dysfunction and apoptosis.^25^ In membranes, oxidative degradation of the phospholipid acyl chains induces reorganization of membrane-soluble free cholesterol into sterol-rich, phospholipid-poor domains, which can act as nucleating sites for proinflammatory cholesterol crystals that trigger NLRP3 inflammasome activity and apoptosis.^21^^,^^26^^,^^27^

We also further investigated the oxidation characteristics of Lp(a)-enriched fractions following a previous study in which we found that Lp(a)-enriched fractions undergo more rapid oxidation than matching enrichments of other atherogenic ApoB particles.^10^ For the first time, we described a nonlinear relationship between the percentage of total ApoB comprised by Lp(a) and the rate of oxidation. Only at elevated levels (>60% total ApoB), the Lp(a) oxidation rate accelerated exponentially. This may indicate that elevated Lp(a) and its oxPL content fuel oxidation of itself and other lipoproteins, especially at the elevated levels associated with increased ASCVD risk. Indeed, highly enriched Lp(a) plasma again underwent more rapid oxidation than a matching enrichment of sdLDL and nonenriched LDL, possibly arising from elevated oxPL content seeding lipoprotein oxidation. This may be particularly important as laboratory measurements of LDL-cholesterol (LDL-C) cannot distinguish between the contributions of LDL-C and Lp(a)-C due to their shared LDL-like structure.^28^ As the LDL-C becomes more enriched with Lp(a)-C, it undergoes exponentially faster rates of lipoprotein oxidation and, potentially, increased proinflammatory effects on ECs and other proatherogenic mechanisms. Whether attenuation of Lp(a) oxidation with *o*-Atorva(m) and/or *p*-Atorva(m) would reverse the previously reported proinflammatory and stress responses in ECs^10^ is unknown and would be a valuable extension of the current study.

Levels of oxidized LDL are strongly associated with ASCVD events, including with severity of acute coronary syndromes and plaque instability.^29^ We previously found that baseline thiobarbituric acid reactive substances and LOOH levels in patients with coronary disease were associated with increased relative risk of major vascular events and procedures.^30^^,^^31^ This association held consistent even after adjusting for inflammatory markers (including C-reactive protein) and other risk factors (including age, LDL, triglycerides, and so on). These analyses indicated that oxidized lipids, characterized as thiobarbituric acid reactive substances and/or LOOH (both of which were measured in our current in vitro experiments), had an independent effect on major vascular events and procedures.

The relationship between statins and Lp(a) is of great interest for physicians and patients given the strong associations of Lp(a) levels with ASCVD risk and the various reports of statin-induced Lp(a) modulation. It is unclear the degree to which statin-induced changes in Lp(a) are a class effect or associated with specific statins, given the divergent results from multiple meta-analyses.^12^^,^^15^ Whether ASCVD risk changes in response to therapeutic-induced changes in Lp(a) is unknown and currently the subject of multiple ongoing phase 3 clinical trials (NCT04023552, NCT05581303). It also remains to be seen whether dramatic Lp(a) reductions, as shown in multiple phase 2 trials,^32^, ^33^, ^34^ induce adverse side effects—such as new onset type 2 diabetes as has been previously suggested^35^—in patients with high-risk. While we await the results of these pivotal trials, it is important to investigate potential risk mitigation therapies for patients with elevated Lp(a).

The findings of the current study suggest a possible mechanism for decreasing an aspect of Lp(a)'s atherogenicity with atorvastatin, which is independent of changes in Lp(a) or LDL levels. We previously found that the omega-3 fatty acid eicosapentaenoic acid (EPA), the active ingredient in icosapent ethyl, showed potent antioxidant activity in Lp(a) and other lipoprotein fractions arising from lipid-centric free radical scavenging by the methylene-interrupted double bonds of the acyl chain.^10^ We then asked whether EPA- or vehicle-treated Lp(a), following exposure to oxidative conditions, differentially affected EC protein expression. We found that vehicle-treated Lp(a) (which underwent significant oxidation before EC treatment) increased expression of stress response and proinflammatory proteins, including heat shock protein 70 and its cochaperone Bcl-2-associated athanogene 3, matrix metalloproteinase-1, and rhotekin. These protein changes were neutralized with EPA-treated Lp(a) (whose oxidation rate was significantly attenuated by EPA), indicating that unprotected Lp(a) undergoes significant oxidation, which in turn elicits various potentially proatherogenic responses from ECs. The results of that study may help explain the reduction in ASCVD events in a subpopulation of REDUCE-IT (Reduction of Cardiovascular Events with Icosapent Ethyl–Intervention Trial) with elevated baseline Lp(a) with icosapent ethyl.^36^

The current study has several important limitations deserving of consideration. First, oxidation characteristics of isolated lipoproteins and model membranes may differ from those in vivo. For the lipoprotein oxidation experiments, we used an exogenous compound (copper sulfate) to initiate oxidative damage. Future studies may use other sources of free radicals, such as glucose or azo compounds, to initiate the oxidation process and characterize rates of lipoprotein oxidation. Additionally, the Lp(a) was isolated from patients with elevated levels but was otherwise healthy. Whether these results extend to lipoproteins isolated from patients with elevated ASCVD risk is unknown. Future studies should directly compare atorvastatin with rosuvastatin in patients with elevated Lp(a) to see if there are any differences in clinical outcomes.

In conclusion, Lp(a) enriched plasma underwent more rapid oxidation as Lp(a) enrichment increased to levels associated with enhanced ASCVD risk. The rate of oxidation of Lp(a) was also more rapid than matching enrichments of nonenriched LDL and even sdLDL. Active atorvastatin metabolites inhibited oxidation of all ApoB particles and cholesterol-enriched membranes in a dose- and time-dependent fashion compared with other statins due to their unique phenoxy group, which facilitates free radical scavenging activity and lipophilicity. The potent antioxidant actions of atorvastatin metabolites may reduce the atherogenicity of Lp(a) in patients with elevated levels beyond changes in its synthesis.

Perspectives **COMPETENCY IN MEDICAL KNOWLEDGE**: Circulating levels of lipoprotein(a) [Lp(a)] are largely determined genetically, and elevated levels have been linked to increased atherosclerotic cardiovascular disease risk. Statins are standard of care for patients with established cardiovascular risk, though their effects on Lp(a) are ambiguous. There are currently no approved therapies to address the atherogenic risk associated with Lp(a). The exact mechanism behind the proatherogenic association with Lp(a) remains to be fully elucidated but may be linked to the high concentration of oxidized phospholipids, which may render Lp(a) particles more susceptible to oxidation than other ApoB-containing particles.

**TRANSLATIONAL OUTLOOK**: As we hypothesized, Lp(a)-enriched plasma underwent more rapid oxidation than matching enrichments of other ApoB-containing lipoproteins, including small dense LDL. We identified a potential antiatherogenic mechanism for multiple active, hydroxylated metabolites of atorvastatin centered around preventing oxidation of Lp(a). This effect was unique to the hydroxylated forms of atorvastatin when compared with other statins in their active form as well as the atorvastatin prodrug. We link this effect to the unique physicochemical properties of the atorvastatin hydroxylated metabolites, which facilitate both membrane penetration and free radical stabilization to attenuate lipoprotein oxidation. The potent antioxidant actions of atorvastatin metabolites may reduce the atherogenicity of Lp(a) in patients with elevated levels beyond changes in its synthesis.

## Funding Support and Author Disclosures

Financial support for this study was provided by an investigator-initiated grant from Viatris with additional funding from Elucida Research. Dr Sherratt is an employee of Elucida Research, which receives research funding from Amarin Pharma Inc., Esperion Therapeutics, Lexicon Pharmaceuticals, and Viatris, and has also received an honorarium from Novartis. Dr Mason receives research funding from Amarin Pharma Inc., Lexicon Pharmaceuticals, Esperion Therapeutics, and Viatris. Dr Bhatt discloses the following relationships - Advisory Board: Angiowave, Antlia Bioscience, Bayer, Boehringer Ingelheim, CellProthera, Cereno Scientific, E-Star Biotech, High Enroll, Janssen, Level Ex, McKinsey, Medscape Cardiology, Merck, NirvaMed, Novo Nordisk, Repair Biotechnologies, Stasys, SandboxAQ (stock options), Tourmaline Bio, Viatris; Board of Directors: American Heart Association New York City, Angiowave (stock options), Bristol Myers Squibb (stock), DRS.LINQ (stock options), High Enroll (stock); Consultant: Alnylam, Altimmune, Broadview Ventures, Corcept Therapeutics, Corsera, GlaxoSmithKline, Hims, Sanofi, SERB, SFJ, Summa Therapeutics, Worldwide Clinical Trials; Data Monitoring Committees: Acesion Pharma, Assistance Publique-Hôpitaux de Paris, Baim Institute for Clinical Research, Boston Scientific (Chair, PEITHO trial), Cleveland Clinic, Contego Medical (Chair, PERFORMANCE 2), Duke Clinical Research Institute, Mayo Clinic, Mount Sinai School of Medicine (for the ABILITY-DM trial, funded by Concept Medical; for ALLAY-HF, funded by Alleviant Medical), Novartis, Population Health Research Institute; Rutgers University (for the NIH-funded MINT Trial); Honoraria: American College of Cardiology (Senior Associate Editor, Clinical Trials and News, ACC.org; Chair, ACC Accreditation Oversight Committee), Arnold and Porter law firm (work related to Sanofi/Bristol-Myers Squibb clopidogrel litigation), Baim Institute for Clinical Research (AEGIS-II executive committee funded by CSL Behring), Belvoir Publications (Editor in Chief, Harvard Heart Letter), Canadian Medical and Surgical Knowledge Translation Research Group (clinical trial steering committees), CSL Behring (AHA lecture), Duke Clinical Research Institute, Engage Health Media, HMP Global (Editor in Chief, Journal of Invasive Cardiology), Medtelligence/ReachMD (CME steering committees), MJH Life Sciences, Oakstone CME (Course Director, Comprehensive Review of Interventional Cardiology), Philips (Becker's Webinar on AI), Population Health Research Institute, WebMD (CME steering committees), Wiley (steering committee); Other: Clinical Cardiology (Deputy Editor, unpaid); Progress in Cardiovascular Diseases (Deputy Editor, unpaid); Added Health (Editorial Board; stock options); Patent: Sotagliflozin (named on a patent for sotagliflozin assigned to Brigham and Women's Hospital who assigned to Lexicon; neither I nor Brigham and Women's Hospital receive any income from this patent); Research Funding: Abbott, Acesion Pharma, Afimmune, Alnylam, Amarin, Amgen, AstraZeneca, Atricure, Bayer, Boehringer Ingelheim, Boston Scientific, CellProthera, Cereno Scientific, Chiesi, Cleerly, CSL Behring, Faraday Pharmaceuticals, Fractyl, Idorsia, Janssen, Javelin, Lexicon, Lilly, Medtronic, Merck, MiRUS, Moderna, Novartis, Novo Nordisk, Pfizer, PhaseBio, Regeneron, Reid Hoffman Foundation, Roche, Sanofi, Stasys, 89Bio; Royalties: Elsevier (Editor, Braunwald’s Heart Disease); Site Co-Investigator: Cleerly. Dr Libby is a consultant and involved in clinical trials for Abcentra, Amgen, DrugFarm, Esperion, Kowa, Novartis, Novo Nordisk, and Ventyx; is a member of scientific advisory boards for Abcentra, Amgen, Novartis, Olatec, Xbiotech, Polygon, and Soley Therapeutics; his laboratory has received research funding from Novartis, Novo Nordisk, and Genentech; is on the Board of Directors of Abcentra; has a financial interest in Xbiotech, a company developing therapeutic human antibodies, TenSixteen Bio, a company targeting somatic mosaicism and clonal hematopoiesis of indeterminate potential (CHIP) to discover and develop novel therapeutics to treat age-related diseases, and Soley Therapeutics, a biotechnology company that is combining artificial intelligence with molecular and cellular response detection for discovering and developing new drugs, currently focusing on cancer therapeutics; and has received funding support from the National Heart, Lung, and Blood Institute (R01HL170000, 1R01HL163099-01, R01AG063839, R01HL151627, R01HL157073, R01HL166538) and the RRM Charitable Fund.
