# Supplementary material for: Rates of Lipoprotein(a) Oxidation Accelerate at Elevated Levels and Attenuate With Atorvastatin Active Metabolites Compared With Other Statins
Source: JACC Basic Transl Sci. 2026 Aug 24;11(9):101664. doi: 10.1016/j.jacbts.2026.101664 (PMC13542599; doi:10.1016/j.jacbts.2026.101664)
Supplement: Supplementary file 1 [file mmc1.docx]

**Supplemental Materials**

**Figure S1.** **Summary of conversion of atorvastatin to its active hydroxylated metabolites.** Approximately 70% of LDL reduction (HMG-CoA reductase inhibition) with atorvastatin is due to the equipotent 2- and 4-hydroxylated metabolites. Structures created using ChemSketch (Freeware) version 2024.2.3 from ACD/Labs.

**Figure S2** **(A) Time-dependent oxidation rate of Lp(a)-enriched lipoprotein fractions compared with sdLDL and LDL fractions; (B) 0.5 h time point.** Lp(a)-enriched fractions exhibited more rapid oxidation than sdLDL- or LDL enriched fractions, including higher baseline oxidized lipid content. At 0.5 h, more than 85% of total lipid is oxidized in Lp(a)-enriched fractions, significantly more than sdLDL or LDL fractions. Statistical analysis: **p<0.05 vs sdLDL; ^§^p<0.05 vs LDL (pairwise Student's t-test). Values are mean ± SD (N = 3).

**Figure S3.** **Dose-dependent effects of o-Atorva(m) on enriched fractions of (A-B) high-enriched Lp(a), (C-D) low-enriched Lp(a), (E-F) sdLDL, and (G-H) LDL.** Statistical indicators – (B) ***p<0.001 vs vehicle; ^‡^p<0.01 vs o-Atorva(m) 1.0 µM. (D) ***p<0.001 vs vehicle; ^§^p<0.001 vs o-Atorva(m) 1.0 µM. (F) ***p<0.001 vs vehicle; ^‡^p<0.001 vs o-Atorva(m) 1.0 µM; ^§^p<0.05 vs o-Atorva(m) 5.0 µM. (H) ***p<0.001 vs vehicle; ^‡^p<0.001 vs o-Atorva(m) 1.0 µM. All tests were performed using Tukey-Kramer multiple comparisons test; overall ANOVA: p<0.001 for each test. Values are mean ± SD (N = 3-5). Individual data points represented by superimposed circles on each bar.

**Figure S4.** **(A) Comparative effects of *o*-Atorva(m) and other statins on LDL oxidation through 4 hours and (B) at the 2 h time point, corresponding to peak MDA levels.** Statistical indicators: ***p<0.001 vs vehicle; ^§^p<0.001 vs other statins (Tukey-Kramer Multiple Comparisons Test; overall ANOVA: p<0.001). Values are mean ± SD (N = 3). Individual data points represented by superimposed circles on each bar.

**Figure S5.** **Separate and Combined Effects of *para*- and *ortho*-Atorvastatin Hydroxy Metabolite on oxidation of lipoprotein fractions enriched with (A-B) high-enriched Lp(a), (C-D) low-enriched Lp(a), (E-F) sdLDL, and (G-H) LDL.** All treatments delivered at the same total statin concentration (10 µM for *p*-Atorva(m) and *o*-Atorva(m), separately or 5 µM of each in the combination treatments). Statistical indicators: (B) *p<0.05 vs vehicle; ***p<0.001 vs vehicle; ^‡^p<0.05 vs *p*-Atorva(m) (overall ANOVA: p = 0.0002); (D) ***p<0.001 vs vehicle; ^‡^p<0.001 vs *p*-Atorva(m) (overall ANOVA: p<0.0001). (F) ***p<0.001 vs vehicle (overall ANOVA: p<0.0001). (H) ***p<0.001 vs vehicle (overall ANOVA: p<0.001). All tests were performed using Tukey-Kramer multiple comparisons test. Values are mean ± SD (N = 3-6). Individual data points represented by superimposed circles on each bar.

**Figure S6.** **Comparative effects of combined hydroxylated atorvastatin metabolites versus other statins in (A) high-enriched Lp(a), (B) low-enriched Lp(a), (C) sdLDL, and (D) LDL.** All treatments were delivered at the same total statin concentration (10 µM total). Values expressed as mean ± SD (N = 3-6).
